# Supplementary material for: ABHD5 blunts the sensitivity of colorectal cancer to fluorouracil via promoting autophagic uracil yield
Source: Nat Commun. 2019 Mar 6;10:1078. doi: 10.1038/s41467-019-08902-x (PMC6403256; doi:10.1038/s41467-019-08902-x)
Supplement: Supplementary file 1 — Supplementary Information [file 41467_2019_8902_MOESM1_ESM.pdf]

ABHD5 Blunts the Sensitivity of Colon Cancer to Fluorouracil via promoting Autophagic Uracil Yield

Juanjuan Ou, *et al.*

Supplementary Figures

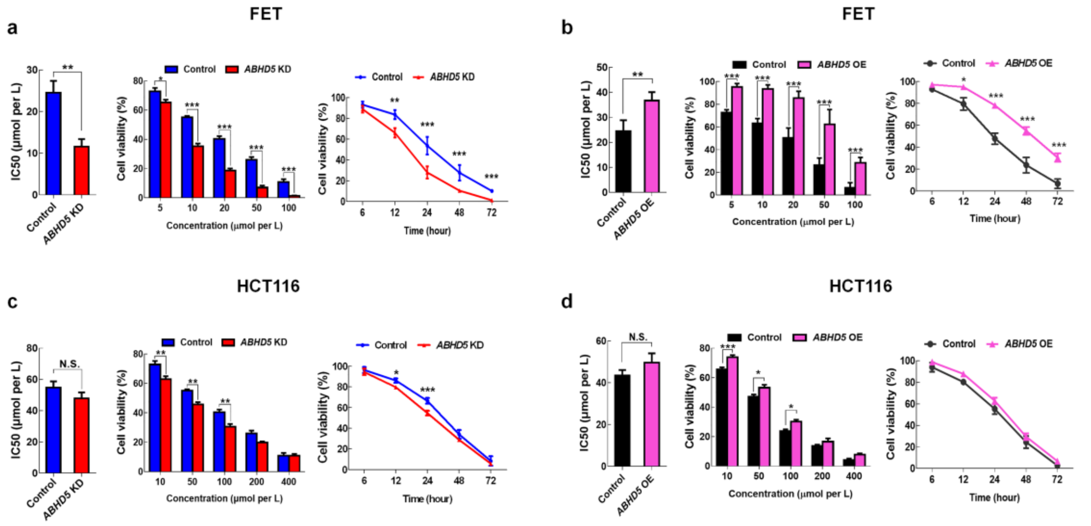

Supplementary Figure 1. ABHD5 exhibits a modest effect on the sensitivity of dMMR cells to FU. (a) The IC50 values of *ABHD5* knockdown (*ABHD5* KD) and control pMMR CRC cells FET to FU were determined by the MTT assay (*n* = 3); *ABHD5* KD and control FET cells were treated with different concentrations of FU for 24 h, and the cell viability was determined by the MTT assay (*n* = 5); *ABHD5* KD and control FET cells were treated with 20 µM FU for different times, and cell viability was determined by the MTT assay (*n* = 4). **(b)** The IC50 values of *ABHD5* overexpression (*ABHD5* OE) and control pMMR CRC cells FET to FU were determined by the MTT assay (*n* = 3); *ABHD5* OE and control FET cells were treated with different concentrations of FU for 24 h, and the cell viability was determined by the MTT assay (*n* = 5); *ABHD5* OE and control FET cells were treated with 20 µM FU for different times, and cell viability was determined by the MTT assay (*n* = 4). **(c)** The IC50

values of *ABHD5* KD and control dMMR CRC cells HCT116 to FU were determined by the MTT assay (n = 3); *ABHD5* KD and control HCT116 cells were treated with different concentrations of FU for 24 h, and the cell viability was determined by the MTT assay (n = 5); *ABHD5* KD and control HCT116 cells were treated with 50 μM FU for different times, and cell viability was determined by the MTT assay (n = 4). **(d)** The IC50 values of *ABHD5* OE and control HCT116 cells to FU were determined by the MTT assay (n = 3); *ABHD5* OE and control HCT116 cells were treated with different concentrations of FU for 24 h, and the cell viability was determined by the MTT assay (n = 5); *ABHD5* OE and control HCT116 cells were treated with 50 μM FU for different times, and cell viability was determined by the MTT assay (n = 4). The quantitative data were presented as mean±S.D (error bar) (*$p < 0.05$, **$p < 0.01$, ***$p < 0.001$, Student's t-test).

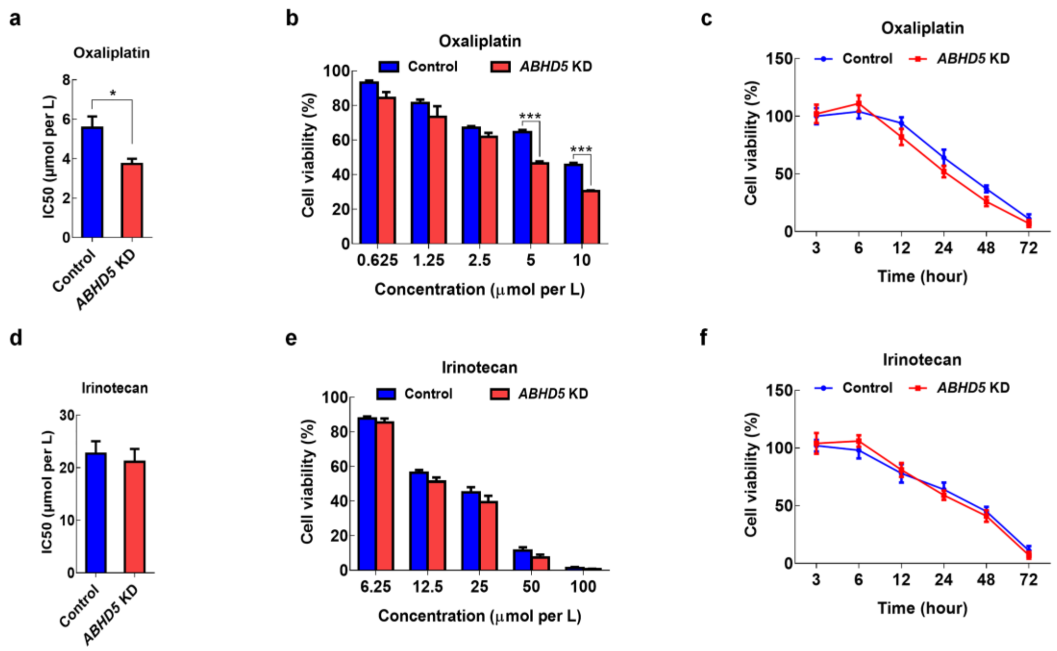

Supplementary Figure 2. ABHD5 modestly affects the sensitivity of CRCs to oxaliplatin and irinotecan. (a) The IC50 values of *ABHD5* KD and control

pMMR CRC cells SW480 to oxaliplatin were determined by the MTT assay (*n* = 3). **(b)** *ABHD5* KD and control SW480 cells were treated with different concentrations of oxaliplatin for 24 h, and cell viability was determined by the MTT assay (*n* = 5). **(c)** *ABHD5* KD and control SW480 cells were treated with 5 μM oxaliplatin for different times, and cell viability was determined by the MTT assay (*n* = 3). **(d)** The IC50 values of *ABHD5* KD and control SW480 cells to irinotecan were determined by the MTT assay (*n* = 3). (**e)** *ABHD5* KD and control SW480 cells were treated with different concentrations of Irinotecan for 24 h, and the cell viability was determined by the MTT assay (*n* = 5). (**f)** *ABHD5* KD and control SW480 cells were treated with 25 μM irinotecan for different times, and cell viability was determined by the MTT assay (*n* = 3). The quantitative data were presented as mean±S.D (error bar) (**p* < 0.05, ****p* < 0.001, Student’ s *t*-test).

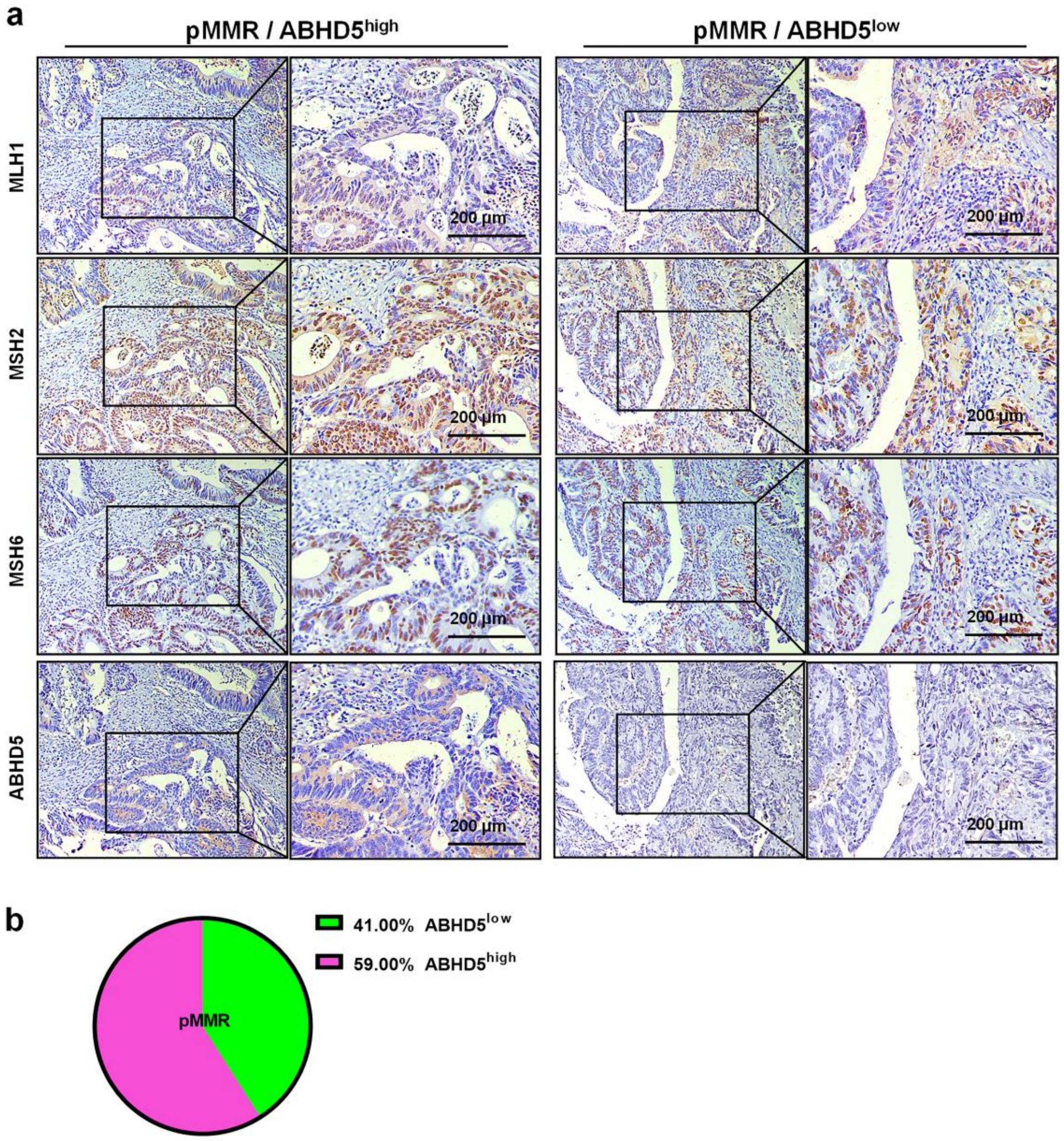

Supplementary Figure 3. The distribution of ABHD5^{low} and ABHD5^{high} subgroups in pMMR CRCs. (a) Immunohistochemistry staining of MLH1, MSH2, MSH6 and ABHD5 stratifying CRCs into subtypes with pMMR/ABHD5^{low} or pMMR/ABHD5^{high} status. Scale bar: 200μm. **(b)** Analysis of the proportions of ABHD5^{low} and ABHD5^{high} subgroups in pMMR CRCs.

a

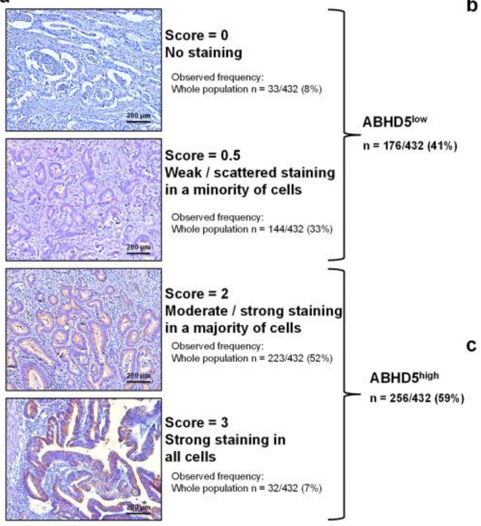

b

Agreement in the final evaluation of the ABHD5 score in individual patients (n = 432) [1]

Final evaluation of patient ABHD5 score (0,0.5,2,3)		Observer #1 Score 0	Score 0.5	Score 2	Score 3
Observer #2	Score 0	30	15	0	0
	Score 0.5	8	124	2	0
	Score 2	0	0	215	4
	Score 3	0	0	4	30

Cohen's Kappa Index With linear weighting
$\kappa = 0.966$
(95% CI = 0.991-0.941)

c

Agreement in the final evaluation of the ABHD5 status in individual patients (n = 432) [2]

Final evaluation of patient status $ABHD5^{low}$ vs. $ABHD5^{high}$		Observer #1 $ABHD5^{low}$	$ABHD5^{high}$
Observer #2	$ABHD5^{low}$	176	0
	$ABHD5^{high}$	0	256

Cohen's Kappa Index With linear weighting
$\kappa = 1$

Supplementary Figure 4. Scoring system for ABHD5 expression using immunohistochemistry. (a) We scored as $ABHD5^{low}$ all tumors with a malignant epithelial component that either completely lacked ABHD5 expression or showed faint cytoplasm expression in a minority of malignant epithelial cells, a feature observed in 41% (*n* = 177/432) of colon carcinomas in our tissue chips. Tumors scored as $ABHD5^{low}$ fell into two staining patterns: complete lack of ABHD5 expression (Score 0), observed in 8% (*n* = 33/432); scattered and faint cytoplasmic expression in a minority of cancer cells (Score 0.5), observed in 33% (*n* = 144/432). We scored as $ABHD5^{high}$ all tumors with a malignant epithelial component that displayed widespread cytoplasmic expression of ABHD5, a feature observed in 59% (*n* = 255/432) of colon carcinomas in tissue chip. Tumors scored as $ABHD5^{high}$ also fell into two staining patterns: strong staining in a majority of cancer cells (Score 2), observed in 52% (*n* = 223/432); strong staining in all cancer cells (Score 3), observed in 7% (*n* = 32/432). For each tumor, two independent tissue cores from distinct areas of the same lesion were analyzed. Tumors with discordant scores on the two cores were upgraded to the highest score. Scale bar: 200µm.

(b) The concordance between the two observers was analyzed using contingency tables to calculate Cohen’s Kappa Index. The results showed excellent agreement ($k > 0.9$). **(c)** The results showed perfect agreement ($k = 1$) with regard to the final assessment of the patients’ ABHD5 status.

a

Pathological Grade	ABHD5 status		% ABHD5^{low}	OR (95% CI)
	ABHD5^{low}	ABHD5^{high}		
G1/G2 (n=348)	217	131	(217/348)	1.12 (1.01-1.24)
G3 (n=84)	41	43	(41/84)	0.64 (0.44-0.94)

Pearson's Chi-squared Test

$\chi^2 = 5.16$

$p = 0.023$

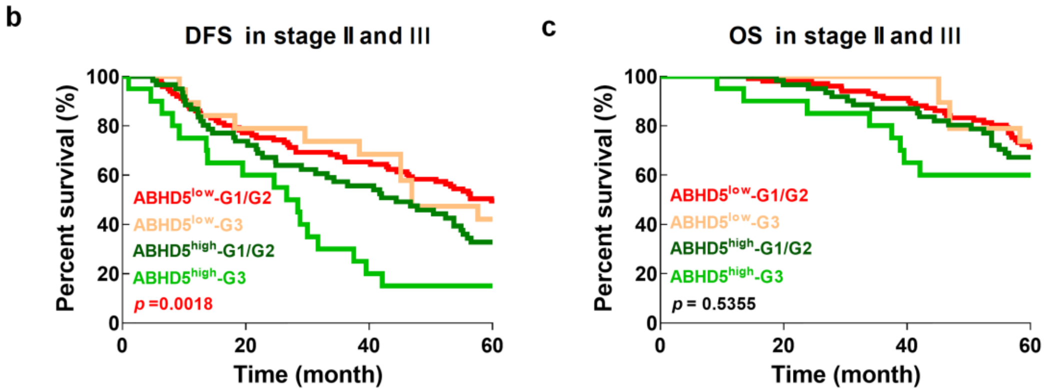

Supplementary Figure 5. Relationship between ABHD5 expression and pathological grade. (a) An analysis of the distribution of low/intermediate grade (G1/G2) vs. high grade (G3) tumors with respect to ABHD5 protein expression in (n = 39) showed that high grade tumors (G3) were enriched in ABHD5^{low} tumors (χ^2-test). (**b, c)** However, the association between ABHD5^{low} tumors and reduced survival appeared to be independent of the pathological grade. ABHD5^{low} tumors with a low/intermediate pathological grade (G1/G2) were characterized by poor clinical outcomes, similar to those observed in ABHD5^{low} tumors with a high pathological grade (G3) and were substantially worse than those observed in ABHD5^{high} tumors, independently of their

low/intermediate or (G1/G2) high (G3) pathological grade. This effect was observed with respect to both 5-year disease-specific survival (DFS) **(b)** and 5-year overall survival (OS) **(c)** (Log-rank test).

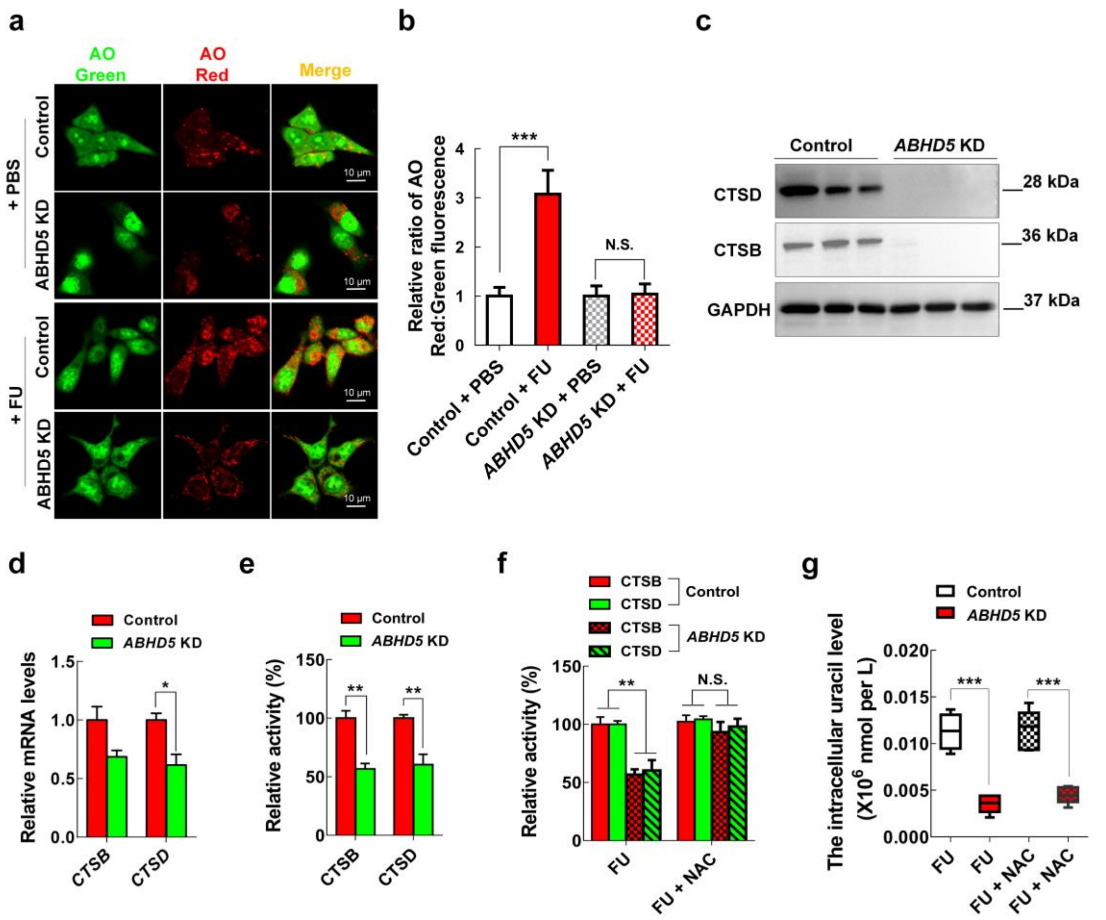

Supplementary Figure 6. Loss of ABHD5 impairs lysosomal activity. (a) ABHD5 affects the AO staining of acidic compartments. *ABHD5* KD or control SW480 cells were treated with or without FU (25 μM) for 6 h before addition of AO. In both control and *ABHD5* KD cells, the cytoplasm and nucleus essentially displayed green fluorescence, whereas the acidic compartments, including the lysosomes, displayed red fluorescence. **(b)** Acidification of the latter compartments was inhibited by loss of ABHD5. The ratio of red to green in the cytosolic region shown in panel **(a)** was calculated. At least 50 cells were

counted, and the ratio of red to green in the cytosolic region was calculated. Scale bar: 10 μm. **(c)** *ABHD5* KD and control SW480 cells were treated with FU (25 μM) for 24 h, and western blots analyses showing the expression levels of CTSB and CTSD in *ABHD5* KD and control SW480 cells. **(d)** *ABHD5* KD and control SW480 cells were treated with FU (25 μM) for 12 h, the relative *CTSB* and *CTSD* mRNA levels (compared with *GAPDH*) in *ABHD5* KD and control SW480 cells were analyzed by quantitative real-time PCR (n = 3). **(e)** *ABHD5* KD and control SW480 cells were treated with FU (25 μM) for 12 h, and the enzymatic activity of CTSB and CTSD was analyzed using fluorogenic kits (n = 3). **(f)** *ABHD5* KD and control SW480 cells were treated with FU (25 μM) or FU (25 μM) + NAC (50 μM) for 6 h, and the enzymatic activities of CTSB and CTSD were analyzed using fluorogenic kits (n = 3). **(g)** *ABHD5* KD and control SW480 cells were treated with FU (25 μM) or FU (25 μM) + NAC (50 μM) for 6 h, and the intracellular uracil was analyzed by LC/MS (n = 3). The quantitative data were presented as mean±S.D (error bar) (N.S., no significance. *$p < 0.05$, **$p < 0.01$, ***$p < 0.001$, Student' s t-test).

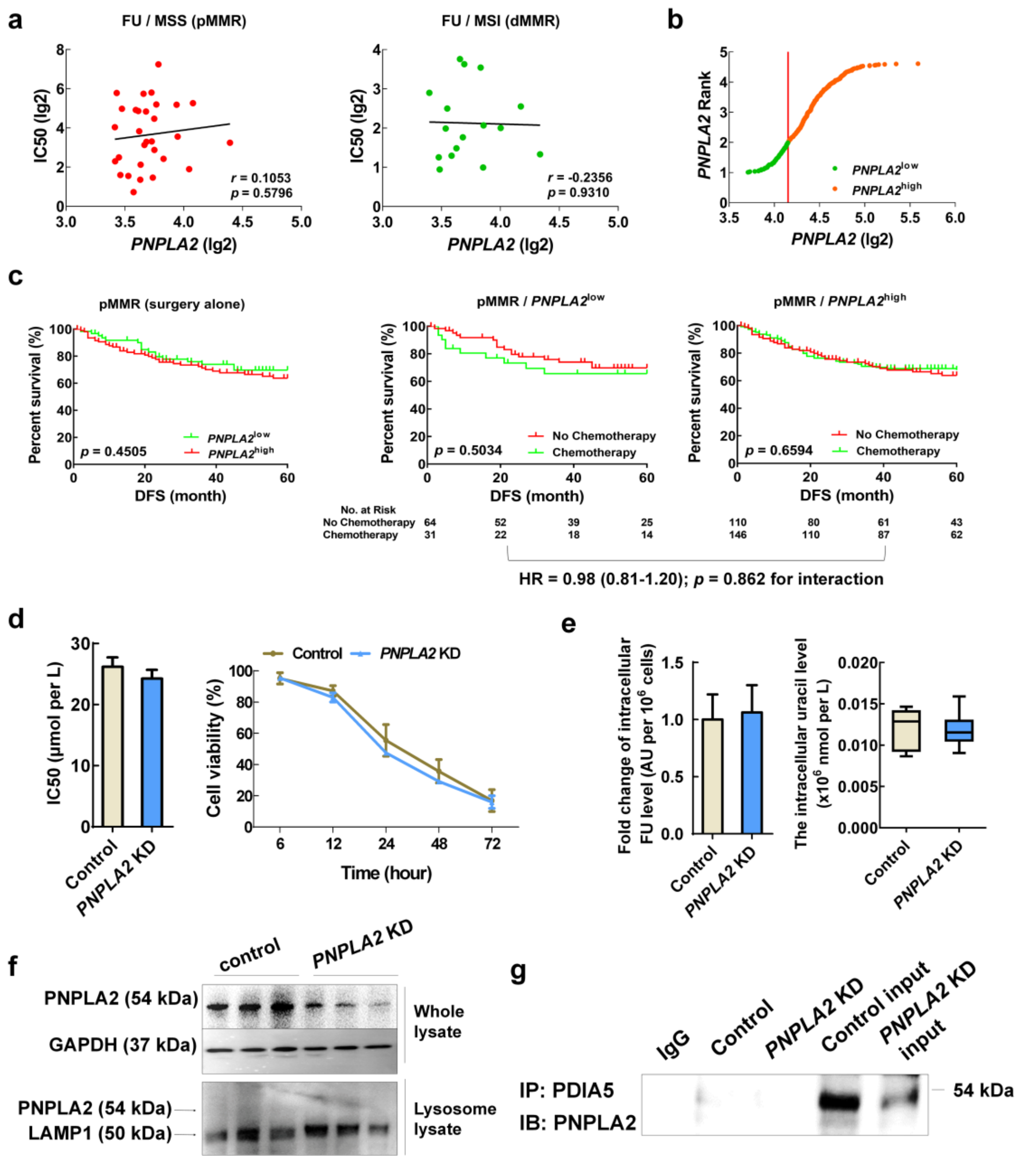

Supplementary Figure 7. ABHD5 regulates CRC response to 5-FU independent of PNPLA2. (a) Analyses from the GDSC dataset revealed the correlation between *PNPLA2* proficiency and the sensitivity to FU in pMMR or dMMR CRC cell lines (*Pearson's* correlations). **(b)** The StepMiner algorithm was used to stratify the population of 361 pMMR CRC patients (stage II/III) in the NCBI-GEO dataset into *PNPLA2*^{high} and *PNPLA2*^{low} subgroups. **(c)** DFS in the subgroup of pMMR/*PNPLA2*^{low} or pMMR/*PNPLA2*^{high} who received surgery alone or treated with or without FU-based adjuvant chemotherapy (Log-rank

test , Cox proportional hazards Regression). **(d)** The IC50 values of *PNPLA2* knockdown (*PNPLA2* KD) and control SW480 cells to FU were determined by the MTT assay (*n* = 3, Student’ s *t*-test). Cell viability of *PNPLA2* KD and control SW480 cells at different time points during challenge with FU (25 μM) were determined by the MTT assay (*n* = 4, Student’ s *t*-test). **(e)** *PNPLA2* KD or control SW480 cells were exposed to PBS or FU (25 μM) for 6 h, and the intracellular concentration of FU was measured by HPLC (*n* = 3, Student’ s *t*-test), and the concentration of intracellular uracil was analyzed by LC/MS(*n* = 6, Student’ s *t*-test). **(f)** The lysosomes were isolated from *PNPLA2* KD and control SW480 cells, and PNPLA2 expression levels in the lysosome lysate and the whole cell lysate were detected by western blots. GAPDH and LAMP1 were used as loading control respectively. **(g)** Immunoprecipitation showing no interaction between PNPLA2 with PDIA5 in *PNPLA2* KD and control SW480 cells. The quantitative data were presented as mean±S.D (error bar).

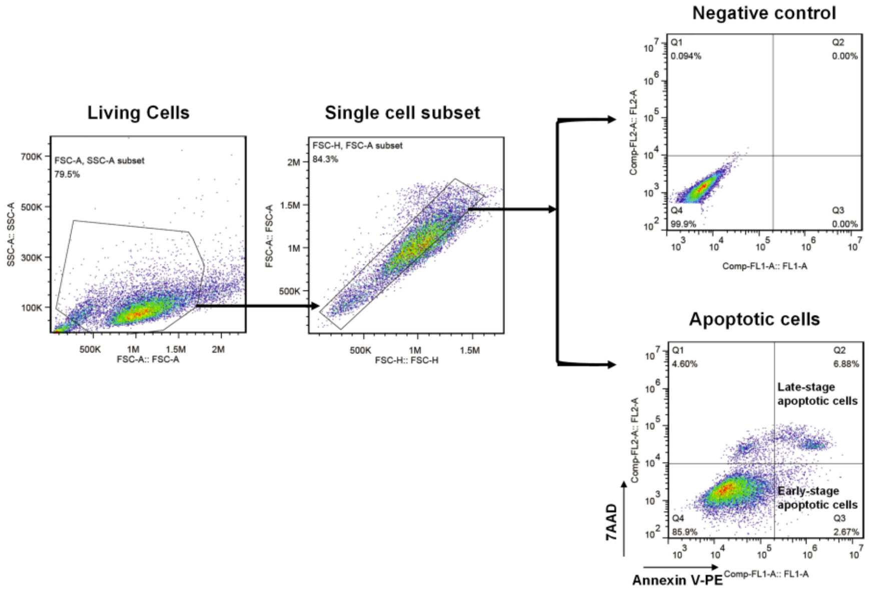

Supplementary Figure 8. Gating strategy to determine the apoptotic rate of CRC cells.

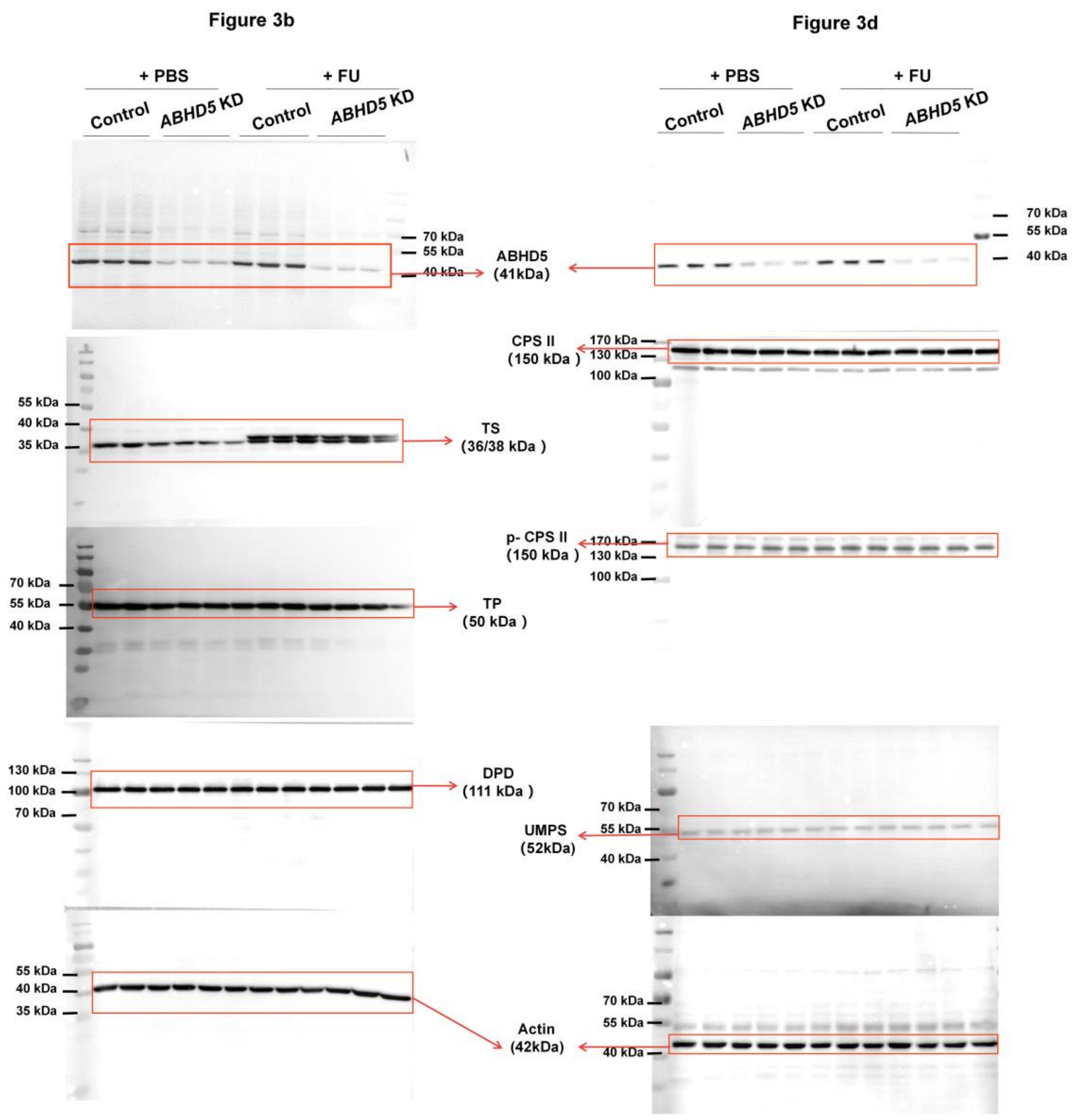

Supplementary Figure 9. The primary images for the cropped blots in Figure 3b and Figure 3d.

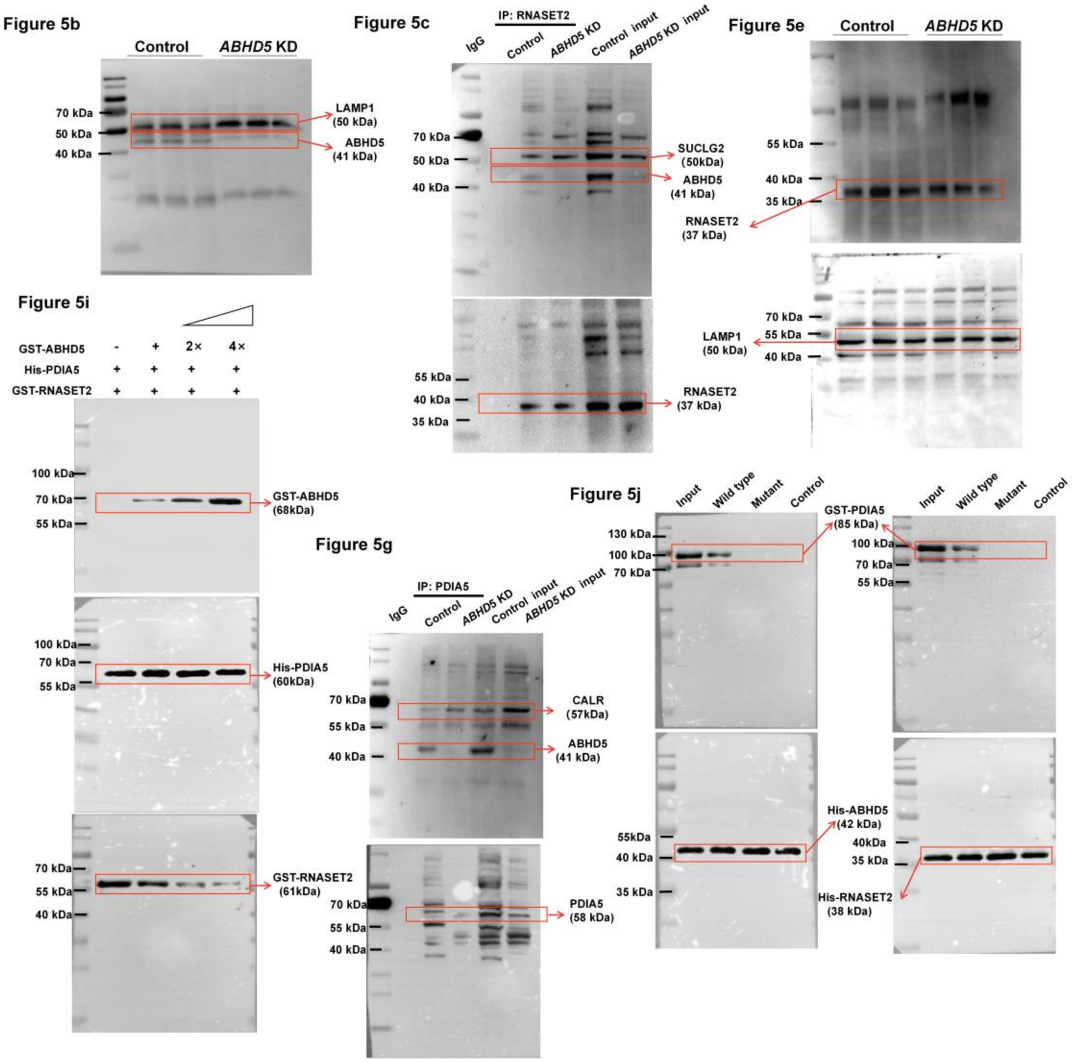

Supplementary Figure 10. The primary images for the cropped blots in Figure 5.

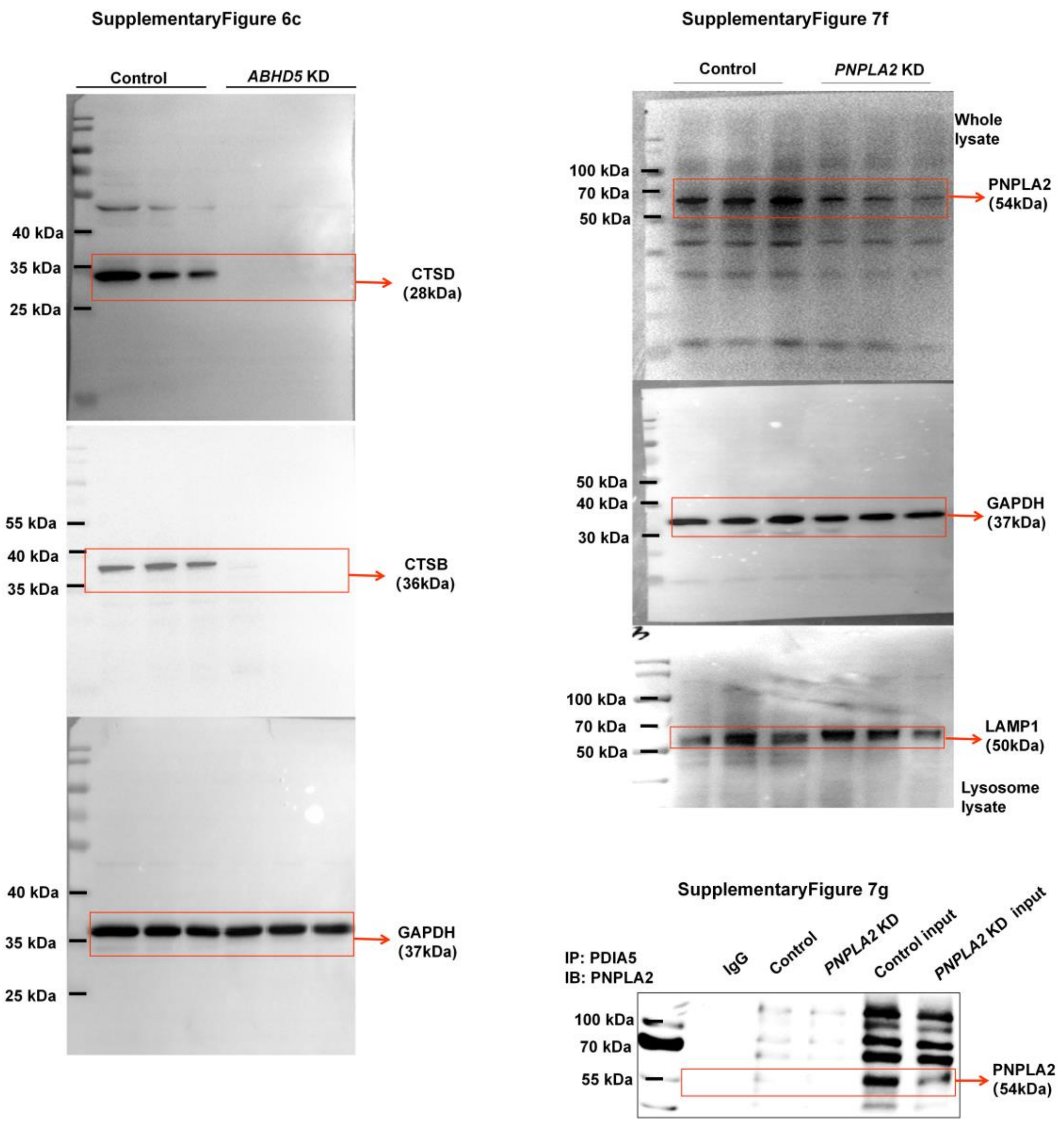

Supplementary Figure 11. The primary images for the cropped blots in Supplementary Figure 6 and Supplementary Figure 7.

Supplementary Table 1. List of Primers Used on This Study

**Target Genes**	**Forward**	**Reverse**
**CTSB**	AGAGTTATGTTTACCGAGGACCT	GATGCAGATCCGGTCAGAGA
**CTSD**	ATTCAGGGCGAGTACATGATCC	CGACACCTTGAGCGTGTAG
